# 1-[3-(4-Chloro­phen­yl)-5-(4-meth­oxy­phen­yl)-4,5-dihydro-1*H*-pyrazol-1-yl]ethanone

**DOI:** 10.1107/S1600536812009439

**Published:** 2012-03-10

**Authors:** Hoong-Kun Fun, Ching Kheng Quah, S. Samshuddin, B. Narayana, B. K. Sarojini

**Affiliations:** a X-ray Crystallography Unit, School of Physics, Universiti Sains Malaysia, 11800 USM, Penang, Malaysia; b Department of Studies in Chemistry, Mangalore University, Mangalagangotri 574 199, India; c Department of Chemistry, P. A. College of Engineering, Nadupadavu, Mangalore 574 153, India

## Abstract

In the title compound, C_18_H_17_ClN_2_O_2_, the benzene rings form dihedral angles of 6.69 (6) and 74.88 (5)° with the 4,5-dihydro-1*H*-pyrazole ring. The benzene rings form a dihedral angle of 76.67 (5)° with each other. In the crystal, mol­ecules are linked *via* bifurcated (C,C)–H⋯O hydrogen bonds into chains along [010]. The crystal structure is further consolidated by C—H⋯π inter­actions.

## Related literature

For general background to and the biological activity of the title compound, see: Samshuddin *et al.* (2011 ▶); Sarojini *et al.* (2010 ▶). For standard bond-length data, see: Allen *et al.* (1987 ▶). For the stability of the temperature controller used in the the data collection, see: Cosier & Glazer (1986 ▶). For a related structure, see: Fun *et al.* (2010 ▶).
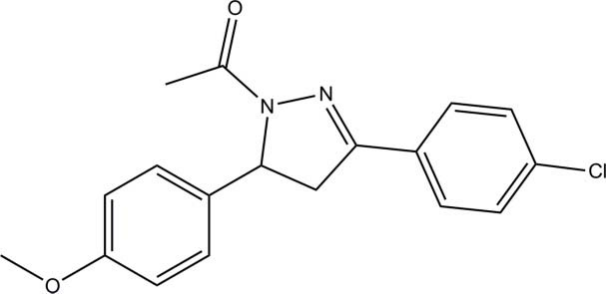


## Experimental

### 

#### Crystal data

C_18_H_17_ClN_2_O_2_
*M* *_r_* = 328.79
Monoclinic, 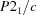

*a* = 9.3473 (4) Å
*b* = 9.4418 (4) Å
*c* = 19.7840 (7) Å
β = 113.830 (2)°
*V* = 1597.19 (11) Å^3^
*Z* = 4
Mo *K*α radiation
μ = 0.25 mm^−1^
*T* = 100 K
0.39 × 0.25 × 0.17 mm

#### Data collection

Bruker SMART APEXII DUO CCD area-detector diffractometer
Absorption correction: multi-scan (*SADABS*; Bruker, 2009 ▶) *T*_min_ = 0.908, *T*_max_ = 0.958
21450 measured reflections
5715 independent reflections
4900 reflections with *I* > 2σ(*I*)
*R*_int_ = 0.020

#### Refinement

*R*[*F* ^2^ > 2σ(*F* ^2^)] = 0.037
*wR*(*F* ^2^) = 0.109
*S* = 1.08
5715 reflections
210 parameters
H-atom parameters constrained
Δρ_max_ = 0.48 e Å^−3^
Δρ_min_ = −0.26 e Å^−3^

### 

Data collection: *APEX2* (Bruker, 2009 ▶); cell refinement: *SAINT* (Bruker, 2009 ▶); data reduction: *SAINT*; program(s) used to solve structure: *SHELXTL* (Sheldrick, 2008 ▶); program(s) used to refine structure: *SHELXTL*; molecular graphics: *SHELXTL*; software used to prepare material for publication: *SHELXTL* and *PLATON* (Spek, 2009 ▶).

## Supplementary Material

Crystal structure: contains datablock(s) global, I. DOI: 10.1107/S1600536812009439/bv2200sup1.cif

Structure factors: contains datablock(s) I. DOI: 10.1107/S1600536812009439/bv2200Isup2.hkl

Supplementary material file. DOI: 10.1107/S1600536812009439/bv2200Isup3.cml

Additional supplementary materials:  crystallographic information; 3D view; checkCIF report

## Figures and Tables

**Table 1:** Hydrogen-bond geometry (Å, °) *Cg*1 is the centroid of C10–C15 benzene ring.

*D*—H⋯*A*	*D*—H	H⋯*A*	*D*⋯*A*	*D*—H⋯*A*
C5—H5*A*⋯O2^i^	0.95	2.55	3.4993 (14)	174
C16—H16*B*⋯O2^ii^	0.98	2.59	3.5275 (12)	161
C16—H16*C*⋯*Cg*1^iii^	0.98	2.69	3.5333 (10)	145

Symmetry codes: (i) 
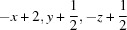
; (ii) 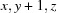; (iii) 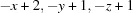.

**Fig. 1.**
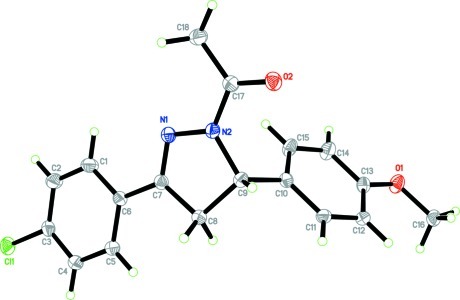
The molecular structure of the title compound showing 50% probability displacement ellipsoids for non-H atoms.

**Fig. 2.**
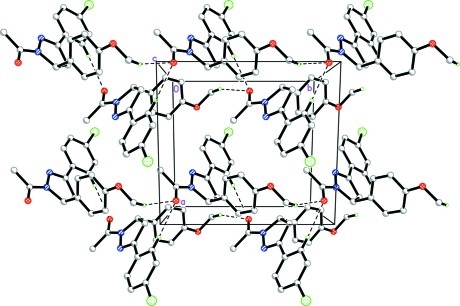
The crystal structure of the title compound, viewed along the c axis. H atoms not involved in hydrogen bonds (dashed lines) have been omitted for clarity.

## supplementary crystallographic information

### Comment

Pyrazolines are know for exhibiting biological properties such as antibacterial, antifungal, antioxidant and analgesic activities (Samshuddin *et al.*, 2011; Sarojini *et al.*, 2010). In continuation of our work on synthesis of pyrazoline derivatives (Fun *et al.*, 2010), the title compound (I) is prepared and its crystal structure is reported.

In the title molecule (Fig. 1), the two benzene rings (C1-C6 and C10-C15) form dihedral angles of 6.69 (6) and 74.88 (5)°, respectively, with the 4,5-dihydro-1*H*-pyrazole ring (N1/N2/C7-C9). The benzene rings form a dihedral angle of 76.67 (5)°. Bond lengths (Allen *et al.*, 1987) and angles are within normal ranges and are comparable with a related structures (Fun *et al.*, 2010).

In the crystal structure, Fig. 2, molecules are linked *via* intermolecular bifurcated C5–H5A···O2 and C16–H16B···O2 hydrogen bonds (Table 1) into one-dimensional chains along [010]. The crystal structure is further consolidated by C16–H16C···Cg1^iii^ (Table 1) interactions, where Cg1 is the centroid of C10-C15 benzene ring.

### Experimental

A mixture of (2*E*)-1-(4-chlorophenyl)-3-(4-methoxyphenyl)prop-2-en-1-one (2.72 g, 0.01 mol) and hydrazine hydrate (0.5 ml, 0.01 mol) in 25 ml acetic acid was refluxed for 6 h. The reaction mixture was cooled and poured into 50 ml ice-cold water. The precipitate was collected by filtration and purified by recrystallization from ethanol. The single crystals were grown from dimethylformamide (DMF) by slow evaporation method and yield of the compound was 82% (*m.p.* : 409 K).

### Refinement

All H atoms were positioned geometrically and refined using a riding model with C–H = 0.95 or 1.00 Å and *U*_iso_(H) = 1.2 or 1.5 *U*_eq_(C). A rotating group model was applied to the methyl groups.

### Crystal data

**Table:**

C_18_H_17_ClN_2_O_2_	*F*(000) = 688
*M**_r_* = 328.79	*D*_x_ = 1.367 Mg m^−^^3^
Monoclinic, *P*2_1_/*c*	Mo *K*α radiation, λ = 0.71073 Å
Hall symbol: -P 2ybc	Cell parameters from 9967 reflections
*a* = 9.3473 (4) Å	θ = 2.4–32.5°
*b* = 9.4418 (4) Å	µ = 0.25 mm^−^^1^
*c* = 19.7840 (7) Å	*T* = 100 K
β = 113.830 (2)°	Block, colourless
*V* = 1597.19 (11) Å^3^	0.39 × 0.25 × 0.17 mm
*Z* = 4	

### Data collection

**Table:**

Bruker SMART APEXII DUO CCD area-detector diffractometer	5715 independent reflections
Radiation source: fine-focus sealed tube	4900 reflections with *I* > 2σ(*I*)
Graphite monochromator	*R*_int_ = 0.020
φ and ω scans	θ_max_ = 32.6°, θ_min_ = 2.3°
Absorption correction: multi-scan (*SADABS*; Bruker, 2009)	*h* = −14→14
*T*_min_ = 0.908, *T*_max_ = 0.958	*k* = −14→14
21450 measured reflections	*l* = −28→30

### Refinement

**Table:**

Refinement on *F*^2^	Primary atom site location: structure-invariant direct methods
Least-squares matrix: full	Secondary atom site location: difference Fourier map
*R*[*F*^2^ > 2σ(*F*^2^)] = 0.037	Hydrogen site location: inferred from neighbouring sites
*wR*(*F*^2^) = 0.109	H-atom parameters constrained
*S* = 1.08	*w* = 1/[σ^2^(*F*_o_^2^) + (0.0578*P*)^2^ + 0.4018*P*] where *P* = (*F*_o_^2^ + 2*F*_c_^2^)/3
5715 reflections	(Δ/σ)_max_ = 0.001
210 parameters	Δρ_max_ = 0.48 e Å^−^^3^
0 restraints	Δρ_min_ = −0.26 e Å^−^^3^

### Special details

**Table:**

Experimental. The crystal was placed in the cold stream of an Oxford Cryosystems Cobra open-flow nitrogen cryostat (Cosier & Glazer, 1986) operating at 100.0 (1) K.
Geometry. All esds (except the esd in the dihedral angle between two l.s. planes) are estimated using the full covariance matrix. The cell esds are taken into account individually in the estimation of esds in distances, angles and torsion angles; correlations between esds in cell parameters are only used when they are defined by crystal symmetry. An approximate (isotropic) treatment of cell esds is used for estimating esds involving l.s. planes.
Refinement. Refinement of F^2^ against ALL reflections. The weighted R-factor wR and goodness of fit S are based on F^2^, conventional R-factors R are based on F, with F set to zero for negative F^2^. The threshold expression of F^2^ > 2sigma(F^2^) is used only for calculating R-factors(gt) etc. and is not relevant to the choice of reflections for refinement. R-factors based on F^2^ are statistically about twice as large as those based on F, and R- factors based on ALL data will be even larger.

### Fractional atomic coordinates and isotropic or equivalent isotropic displacement parameters (Å^2^)

**Table:**

	*x*	*y*	*z*	*U*_iso_*/*U*_eq_	
Cl1	0.39017 (3)	0.39544 (3)	−0.167559 (12)	0.02538 (8)	
O1	0.79766 (9)	0.66995 (8)	0.45133 (4)	0.02433 (16)	
O2	0.91233 (9)	0.03965 (8)	0.36842 (4)	0.02099 (15)	
N1	0.71059 (10)	0.14269 (8)	0.18233 (4)	0.01673 (15)	
N2	0.81942 (10)	0.13711 (8)	0.25551 (4)	0.01686 (15)	
C1	0.52427 (13)	0.20907 (12)	0.02990 (5)	0.02301 (19)	
H1A	0.4856	0.1381	0.0522	0.028*	
C2	0.44026 (13)	0.24407 (12)	−0.04388 (5)	0.0241 (2)	
H2A	0.3442	0.1979	−0.0721	0.029*	
C3	0.49847 (12)	0.34738 (10)	−0.07580 (5)	0.01920 (18)	
C4	0.63933 (13)	0.41499 (11)	−0.03629 (5)	0.02137 (19)	
H4A	0.6787	0.4838	−0.0594	0.026*	
C5	0.72232 (12)	0.38032 (10)	0.03796 (5)	0.01943 (18)	
H5A	0.8182	0.4269	0.0659	0.023*	
C6	0.66539 (11)	0.27739 (10)	0.07168 (5)	0.01637 (16)	
C7	0.75373 (11)	0.24300 (9)	0.14994 (5)	0.01598 (16)	
C8	0.90463 (11)	0.31439 (10)	0.19905 (5)	0.01772 (17)	
H8A	0.8937	0.4188	0.1971	0.021*	
H8B	0.9915	0.2874	0.1852	0.021*	
C9	0.93042 (11)	0.25651 (9)	0.27597 (5)	0.01613 (16)	
H9A	1.0400	0.2209	0.3019	0.019*	
C10	0.89448 (11)	0.36484 (10)	0.32356 (5)	0.01551 (16)	
C11	1.00985 (11)	0.46168 (10)	0.36358 (5)	0.01698 (16)	
H11A	1.1092	0.4567	0.3612	0.020*	
C12	0.98297 (11)	0.56594 (10)	0.40712 (5)	0.01733 (16)	
H12A	1.0633	0.6309	0.4343	0.021*	
C13	0.83702 (12)	0.57364 (10)	0.41030 (5)	0.01832 (17)	
C14	0.71987 (12)	0.47712 (12)	0.37019 (6)	0.0233 (2)	
H14A	0.6203	0.4822	0.3723	0.028*	
C15	0.74886 (12)	0.37390 (11)	0.32730 (5)	0.02067 (18)	
H15A	0.6687	0.3088	0.3002	0.025*	
C16	0.91421 (13)	0.77111 (10)	0.49274 (5)	0.02186 (19)	
H16A	0.8761	0.8270	0.5238	0.033*	
H16B	0.9360	0.8341	0.4587	0.033*	
H16C	1.0103	0.7214	0.5240	0.033*	
C17	0.81439 (11)	0.03714 (10)	0.30430 (5)	0.01726 (17)	
C18	0.68751 (13)	−0.07237 (11)	0.27532 (6)	0.02370 (19)	
H18A	0.7054	−0.1461	0.3127	0.036*	
H18B	0.6885	−0.1149	0.2303	0.036*	
H18C	0.5858	−0.0276	0.2640	0.036*	

### Atomic displacement parameters (Å^2^)

**Table:**

	*U*^11^	*U*^22^	*U*^33^	*U*^12^	*U*^13^	*U*^23^
Cl1	0.02990 (14)	0.02625 (13)	0.01582 (11)	−0.00074 (9)	0.00491 (9)	0.00026 (8)
O1	0.0228 (4)	0.0255 (3)	0.0257 (3)	−0.0018 (3)	0.0108 (3)	−0.0105 (3)
O2	0.0233 (4)	0.0204 (3)	0.0170 (3)	0.0018 (3)	0.0058 (3)	0.0015 (2)
N1	0.0169 (4)	0.0175 (3)	0.0150 (3)	0.0008 (3)	0.0056 (3)	0.0001 (3)
N2	0.0181 (4)	0.0164 (3)	0.0146 (3)	−0.0012 (3)	0.0051 (3)	−0.0002 (2)
C1	0.0202 (5)	0.0271 (5)	0.0207 (4)	−0.0054 (4)	0.0071 (4)	0.0032 (3)
C2	0.0200 (5)	0.0292 (5)	0.0198 (4)	−0.0060 (4)	0.0047 (4)	0.0012 (3)
C3	0.0215 (5)	0.0202 (4)	0.0153 (4)	0.0008 (3)	0.0068 (3)	−0.0005 (3)
C4	0.0254 (5)	0.0214 (4)	0.0176 (4)	−0.0040 (4)	0.0090 (4)	0.0008 (3)
C5	0.0201 (4)	0.0203 (4)	0.0180 (4)	−0.0038 (3)	0.0078 (3)	−0.0005 (3)
C6	0.0166 (4)	0.0176 (4)	0.0159 (3)	0.0003 (3)	0.0076 (3)	0.0000 (3)
C7	0.0157 (4)	0.0169 (4)	0.0163 (3)	0.0006 (3)	0.0074 (3)	−0.0010 (3)
C8	0.0177 (4)	0.0199 (4)	0.0168 (4)	−0.0027 (3)	0.0083 (3)	−0.0015 (3)
C9	0.0153 (4)	0.0161 (4)	0.0167 (3)	−0.0002 (3)	0.0061 (3)	−0.0010 (3)
C10	0.0157 (4)	0.0165 (4)	0.0135 (3)	0.0008 (3)	0.0050 (3)	0.0006 (3)
C11	0.0165 (4)	0.0173 (4)	0.0182 (4)	−0.0014 (3)	0.0081 (3)	0.0002 (3)
C12	0.0179 (4)	0.0164 (4)	0.0172 (4)	−0.0030 (3)	0.0066 (3)	−0.0008 (3)
C13	0.0194 (4)	0.0190 (4)	0.0165 (4)	0.0009 (3)	0.0072 (3)	−0.0022 (3)
C14	0.0156 (4)	0.0286 (5)	0.0261 (4)	−0.0016 (4)	0.0089 (4)	−0.0089 (4)
C15	0.0150 (4)	0.0241 (4)	0.0215 (4)	−0.0018 (3)	0.0058 (3)	−0.0068 (3)
C16	0.0276 (5)	0.0175 (4)	0.0183 (4)	−0.0010 (4)	0.0070 (4)	−0.0021 (3)
C17	0.0188 (4)	0.0153 (4)	0.0185 (4)	0.0024 (3)	0.0085 (3)	0.0005 (3)
C18	0.0255 (5)	0.0211 (4)	0.0227 (4)	−0.0043 (4)	0.0079 (4)	0.0014 (3)

### Geometric parameters (Å, º)

**Table:**

Cl1—C3	1.7440 (10)	C8—H8A	0.9900
O1—C13	1.3647 (11)	C8—H8B	0.9900
O1—C16	1.4314 (12)	C9—C10	1.5171 (12)
O2—C17	1.2286 (11)	C9—H9A	1.0000
N1—C7	1.2957 (12)	C10—C11	1.3913 (13)
N1—N2	1.3942 (11)	C10—C15	1.3952 (13)
N2—C17	1.3644 (12)	C11—C12	1.3962 (13)
N2—C9	1.4738 (12)	C11—H11A	0.9500
C1—C2	1.3894 (14)	C12—C13	1.3927 (14)
C1—C6	1.3994 (14)	C12—H12A	0.9500
C1—H1A	0.9500	C13—C14	1.3990 (14)
C2—C3	1.3870 (14)	C14—C15	1.3890 (13)
C2—H2A	0.9500	C14—H14A	0.9500
C3—C4	1.3857 (14)	C15—H15A	0.9500
C4—C5	1.3951 (13)	C16—H16A	0.9800
C4—H4A	0.9500	C16—H16B	0.9800
C5—C6	1.3998 (13)	C16—H16C	0.9800
C5—H5A	0.9500	C17—C18	1.5017 (14)
C6—C7	1.4668 (12)	C18—H18A	0.9800
C7—C8	1.5102 (13)	C18—H18B	0.9800
C8—C9	1.5414 (12)	C18—H18C	0.9800
			
C13—O1—C16	117.34 (8)	C10—C9—H9A	110.2
C7—N1—N2	107.39 (8)	C8—C9—H9A	110.2
C17—N2—N1	122.80 (8)	C11—C10—C15	118.56 (8)
C17—N2—C9	123.82 (8)	C11—C10—C9	118.76 (8)
N1—N2—C9	113.18 (7)	C15—C10—C9	122.66 (8)
C2—C1—C6	120.58 (9)	C10—C11—C12	121.53 (9)
C2—C1—H1A	119.7	C10—C11—H11A	119.2
C6—C1—H1A	119.7	C12—C11—H11A	119.2
C3—C2—C1	119.10 (9)	C13—C12—C11	119.24 (8)
C3—C2—H2A	120.4	C13—C12—H12A	120.4
C1—C2—H2A	120.4	C11—C12—H12A	120.4
C4—C3—C2	121.69 (9)	O1—C13—C12	124.47 (9)
C4—C3—Cl1	119.14 (7)	O1—C13—C14	115.73 (9)
C2—C3—Cl1	119.17 (8)	C12—C13—C14	119.79 (9)
C3—C4—C5	118.89 (9)	C15—C14—C13	120.16 (9)
C3—C4—H4A	120.6	C15—C14—H14A	119.9
C5—C4—H4A	120.6	C13—C14—H14A	119.9
C4—C5—C6	120.53 (9)	C14—C15—C10	120.71 (9)
C4—C5—H5A	119.7	C14—C15—H15A	119.6
C6—C5—H5A	119.7	C10—C15—H15A	119.6
C1—C6—C5	119.19 (8)	O1—C16—H16A	109.5
C1—C6—C7	121.05 (8)	O1—C16—H16B	109.5
C5—C6—C7	119.77 (8)	H16A—C16—H16B	109.5
N1—C7—C6	121.93 (8)	O1—C16—H16C	109.5
N1—C7—C8	113.87 (8)	H16A—C16—H16C	109.5
C6—C7—C8	124.15 (8)	H16B—C16—H16C	109.5
C7—C8—C9	102.11 (7)	O2—C17—N2	119.53 (9)
C7—C8—H8A	111.3	O2—C17—C18	123.48 (9)
C9—C8—H8A	111.3	N2—C17—C18	116.99 (8)
C7—C8—H8B	111.3	C17—C18—H18A	109.5
C9—C8—H8B	111.3	C17—C18—H18B	109.5
H8A—C8—H8B	109.2	H18A—C18—H18B	109.5
N2—C9—C10	112.23 (8)	C17—C18—H18C	109.5
N2—C9—C8	100.85 (7)	H18A—C18—H18C	109.5
C10—C9—C8	112.84 (7)	H18B—C18—H18C	109.5
N2—C9—H9A	110.2		
			
C7—N1—N2—C17	−175.94 (8)	N1—N2—C9—C8	−15.47 (9)
C7—N1—N2—C9	9.10 (10)	C7—C8—C9—N2	14.77 (9)
C6—C1—C2—C3	0.28 (17)	C7—C8—C9—C10	−105.14 (8)
C1—C2—C3—C4	0.99 (16)	N2—C9—C10—C11	162.70 (8)
C1—C2—C3—Cl1	−177.99 (8)	C8—C9—C10—C11	−84.18 (10)
C2—C3—C4—C5	−1.61 (16)	N2—C9—C10—C15	−19.12 (12)
Cl1—C3—C4—C5	177.37 (8)	C8—C9—C10—C15	94.01 (11)
C3—C4—C5—C6	0.97 (15)	C15—C10—C11—C12	0.32 (14)
C2—C1—C6—C5	−0.89 (16)	C9—C10—C11—C12	178.58 (8)
C2—C1—C6—C7	178.96 (10)	C10—C11—C12—C13	−0.32 (14)
C4—C5—C6—C1	0.25 (15)	C16—O1—C13—C12	1.13 (14)
C4—C5—C6—C7	−179.59 (9)	C16—O1—C13—C14	−179.58 (9)
N2—N1—C7—C6	179.53 (8)	C11—C12—C13—O1	179.42 (9)
N2—N1—C7—C8	2.14 (10)	C11—C12—C13—C14	0.16 (14)
C1—C6—C7—N1	4.38 (14)	O1—C13—C14—C15	−179.34 (9)
C5—C6—C7—N1	−175.78 (9)	C12—C13—C14—C15	−0.01 (16)
C1—C6—C7—C8	−178.50 (9)	C13—C14—C15—C10	0.02 (16)
C5—C6—C7—C8	1.34 (14)	C11—C10—C15—C14	−0.17 (15)
N1—C7—C8—C9	−11.45 (10)	C9—C10—C15—C14	−178.36 (9)
C6—C7—C8—C9	171.22 (8)	N1—N2—C17—O2	−178.73 (8)
C17—N2—C9—C10	−70.02 (11)	C9—N2—C17—O2	−4.31 (14)
N1—N2—C9—C10	104.88 (8)	N1—N2—C17—C18	1.89 (13)
C17—N2—C9—C8	169.63 (9)	C9—N2—C17—C18	176.31 (8)

### Hydrogen-bond geometry (Å, º)

Cg1 is the centroid of C10–C15 benzene ring.

**Table:**

*D*—H···*A*	*D*—H	H···*A*	*D*···*A*	*D*—H···*A*
C5—H5*A*···O2^i^	0.95	2.55	3.4993 (14)	174
C16—H16*B*···O2^ii^	0.98	2.59	3.5275 (12)	161
C16—H16*C*···*Cg*1^iii^	0.98	2.69	3.5333 (10)	145

Symmetry codes: (i) −*x*+2, *y*+1/2, −*z*+1/2; (ii) *x*, *y*+1, *z*; (iii) −*x*+2, −*y*+1, −*z*+1.
